# A Sketch of the Early History of Practical Anatomy

**Published:** 1870-12

**Authors:** 


A Sketch of the Early History of Practical Anatomy. By W. W. Keen, M.D., Lecturer on Anatomy and Operative Surgery, Philadelphia School of Anatomy; Lecturer on Pathological Anatomy in Jefferson Medical College.

This is a peculiarly happy, interesting and instructive pamphlet, presented in the form of an introductory address. The writer calls us back to the first efforts to advance the science of Anatomy, which was in the city of Alexandria, about 300 years before Christ, Egypt then being the medical centre for the whole world. All the great men of the world repaired to Egypt for instruction. Galen went from Rome to Egypt to see a human skeleton, the practise of burning the bodies at Rome prohibiting the study of practical anatomy on the human subject. With the fall of Alexandria, the science of Anatomy declined, and was not known for a period of twelve centuries, when it was again revived in Italy, at the city of Bologne.

Italy now became the centre of learning for the whole world, and at the famous University of Bologne, there were 10,000

students assembled; here the first modern dissection was performed. Notwithstanding the fame which the Italian schools had acquired, they, also, declined. And, with the sixteenth century, came a new impulse. Vesalius became teacher of Anatomy in Padua, in 1537, and from then we have, successively, Columbus, Eustachius, Falopius, Fabricus, Gassier, Ingrassias, Aræetius, Vesalius, Varolius, and the names of many other distinguished anatomists. The author tells us of many of the difficulties with which the Eastern anatomists had to contend, and of the total ignorance of many professed physicians of the science. He traces the history of dissections down from Italy to England, and giving its history in England, Scotland, and Holland, alluding to all the distinguished anatomists of those days and countries. The limits of a review render it impossible to do justice to the merits of this valuable essay. It is to be lamented that no work on the history of Anatomy has yet appeared in American medical literature, and, for this fact, the essay of Dr. Keen is peculiarly valuable, as including many facts relative to the history of the science, carefully gathered and arranged. H. W. B.

